# Why Use Position Features in Liver Segmentation Performed by Convolutional Neural Network

**DOI:** 10.3389/fphys.2021.734217

**Published:** 2021-10-01

**Authors:** Miroslav Jiřík, Filip Hácha, Ivan Gruber, Richard Pálek, Hynek Mírka, Milos Zelezny, Václav Liška

**Affiliations:** ^1^Department of Cybernetics, Faculty of Applied Sciences, University of West Bohemia, Pilsen, Czechia; ^2^New Technologies for the Information Society, Faculty of Applied Sciences, University of West Bohemia, Pilsen, Czechia; ^3^Biomedical Center, Faculty of Medicine in Pilsen, Charles University, Pilsen, Czechia; ^4^Department of Informatics, Faculty of Applied Sciences, University of West Bohemia, Pilsen, Czechia; ^5^Department of Surgery, University Hospital and Faculty of Medicine in Pilsen, Charles University, Pilsen, Czechia; ^6^Department of Radiology, University Hospital and Faculty of Medicine in Pilsen, Charles University, Pilsen, Czechia

**Keywords:** liver volumetry, semantic segmentation, machine learning, convolutional neural network, medical imaging, position features

## Abstract

Liver volumetry is an important tool in clinical practice. The calculation of liver volume is primarily based on Computed Tomography. Unfortunately, automatic segmentation algorithms based on handcrafted features tend to leak segmented objects into surrounding tissues like the heart or the spleen. Currently, convolutional neural networks are widely used in various applications of computer vision including image segmentation, while providing very promising results. In our work, we utilize robustly segmentable structures like the spine, body surface, and sagittal plane. They are used as key points for position estimation inside the body. The signed distance fields derived from these structures are calculated and used as an additional channel on the input of our convolutional neural network, to be more specific U-Net, which is widely used in medical image segmentation tasks. Our work shows that this additional position information improves the results of the segmentation. We test our approach in two experiments on two public datasets of Computed Tomography images. To evaluate the results, we use the Accuracy, the Hausdorff distance, and the Dice coefficient. Code is publicly available at: https://gitlab.com/hachaf/liver-segmentation.git.

## 1. Introduction

3D medical imaging methods are an essential tool in diagnostics and treatment strategy selection. Magnetic Resonance Imaging is available in all major hospitals, and the Computed Tomography (CT) is available even in smaller ones. The analysis of the image data is traditionally done by the expert—radiologist. During this procedure, the 3D data are usually viewed on a 2D monitor as slices. This makes the process complicated and time-consuming, even for a trained operator.

Semantic segmentation is a task where each pixel in an input image is classified into a specific class, i.e., per-pixel image classification. In the previous two decades, along with developments in the field of computer vision, semi-automatic and automatic liver segmentation tools based on traditional computer vision have been proposed (Moghbel et al., 2018) and many complex Computer-Aided diagnostic systems were introduced (Christ et al., 2017). However, despite all the effort, the problem is not been perfectly solved till today. The main problem is that in a CT image, the intensity of the liver is similar to the intensity of adjacent tissues. Therefore, solely based on the intensity, it is impossible to decide which tissue belongs to the liver and which belongs to another organ. Another challenge is the variability of the liver shape.

Since 2012, when AlexNet by Krizhevsky et al. (2012) dominated ImageNet challenge (Deng et al., 2009), the popularity of the traditional computer vision methods decreases at expenses of neural networks. Most of the recent approaches for semantic segmentation are based on Convolutional Neural Networks (CNNs) (for example, Long et al., 2015; Chen et al., 2017a,b), nevertheless, from 2020 Transformer (Vaswani et al., 2017; Dosovitskiy et al., 2020) based architecture become increasingly popular (for example, Carion et al., 2020; Zheng et al., 2021). Their main disadvantages are the necessity of big amount of training data, and their high computational complexity.

In medical imaging, U-Net (Ronneberger et al., 2015) is the golden standard architecture for semantic segmentation. A nested version of U-Net utilizes authors in Zhou et al. (2018). In the work (Radiuk, 2020), the author proposes 3D U-Net to perform multi-organ segmentation. In recent papers, the authors also experiment with transformers (Valanarasu et al., 2021), and their combination with the U-Net architecture (Chen et al., 2021). In our work, we decided to utilize the standard U-Net architecture with some modifications tailored specifically to our task.

The standard procedure in machine learning is the normalization of the data. Removing the data variability by normalization makes learning more straightforward because the algorithm can be focussed on the simpler problem. In image processing, the most common normalization algorithms are oriented on the object's intensity, size, or position. In CT images, the position normalization is done by the standard pose of the patient, while the calibration of the device provides the intensity normalization.

In this paper, we take a step beyond the limit of the standard CT normalization. We focus on the use of robustly segmentable tissues in the human body, and we show that the precise knowledge of the position of these structures can be used for a relative position determination in the body. Furthermore, this information can be used with benefit for the automatic segmentation of the liver.

The typical size of an abdominal CT image is about 512 × 512 × 100, which gives us 26.214.400 voxels. It is hardly possible to train a neural network with such massive input. The usual way to solve this problem is to split the input image into smaller parts. The two most common attempts to solve this problem are separating the 2D slices or splitting the 3D input image into smaller blocks. However, by this procedure, some information is lost by the cropping operation. Each small slice or block of image data contains essential information about the surrounding tissues, but the position context is missing.

We suggest inserting additional position information into the training procedure. In our work, we used an adapted algorithm from the *bodynavigation* Python package (Jirik and Liska, 2018) to extract the robustly segmentable tissues and the Signed Distance Field (SDF) to each of these segmentations. During the training procedure, The SDFs are attached to the intensity image as additional channels. We tested this setup for two different segmentation approaches and show that it improves the segmentation results for both of them.

## 2. Proposed Method

Our segmentation method is based on a convolutional neural network with U-Net architecture. In this work, we tested two different approaches. The first approach is based on processing a single 2D slice from the original 3D volumetric image using standard U-Net architecture. This approach uses a discrete convolution of two-dimensional signals. Volumetric image is processed slice by slice, and the individual solutions are then assembled back into the original 3D image.

The second approach is based on the use of 3D signal convolution. The volumetric input image is cut into cubes of size 32 × 32 × 32 voxels to overcome memory limits. These cubes are then processed by the neural network and then assembled back into the original 3D image once again. When preparing the training data, these cubes were cut using an offset of 8, 16, and 24 voxels in each direction, significantly increasing the amount of training data. For this approach, all 2D convolution layers in U-Net are replaced by 3D convolution layers, as well as max-pooling layers. The rest of the architecture remains the same.

**Figure 1:**
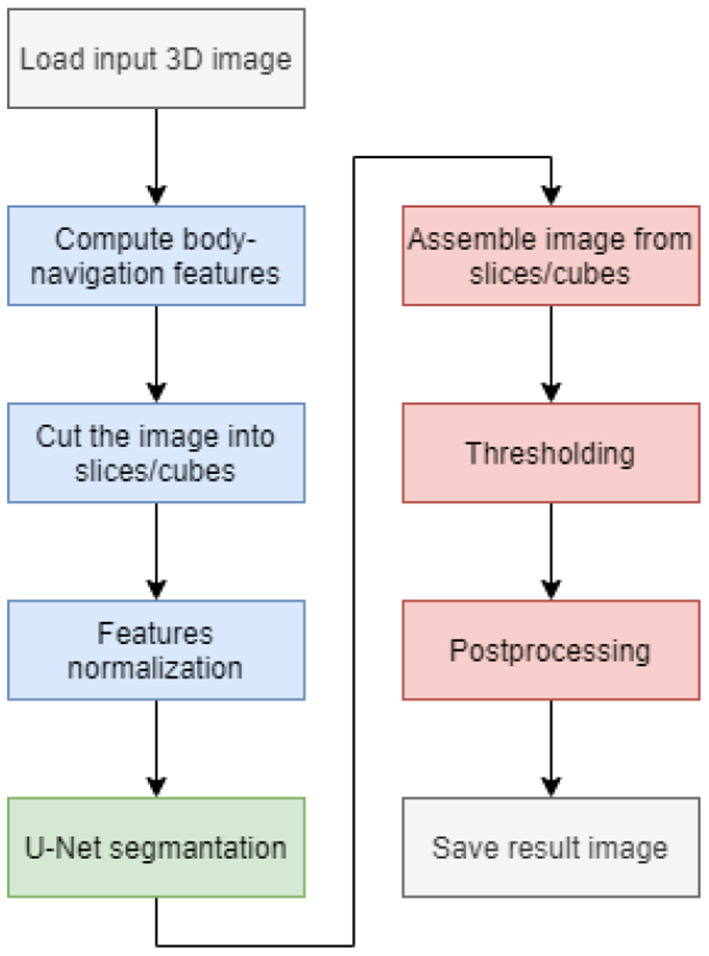
Flowchart of the segmentation process.

In addition to the neural network, the entire segmentation process is complemented by data normalization, feature space expansion using body-navigation features, and postprocessing of neural network output. The diagram of the whole process is shown in Figure 1.

### 2.1. Data Normalization

Before the segmentation itself, we need to normalize the input CT image. The measured X-ray intensities given by the absorption of various tissue types are already partially normalized at the output of the CT scanner. Therefore, we only rescale them to the interval [0, 1] using standard min-max normalization (see Equation 1). The same normalization method is applied to the body-navigation features.


(1) $$\begin{array}{r} x_{norm}=\frac{x-x_{min}}{x_{max}-x_{min}} \end{array}$$

### 2.2. U-Net

The U-Net architecture consists of a contracting path and an expansive path. The contracting path is formed by repeating convolutional layers, followed by a rectified linear unit (ReLU) and a max-pooling layer for downsampling. The expansive path consists of an upsampling of the feature map followed by a convolution layer, a concatenation with the correspondingly cropped feature map from the contracting path, and two convolution layers, each followed by a ReLU. To avoid overfitting the network, we add dropout layers after the fourth and fifth levels of the contracting path.

**Figure 2:**
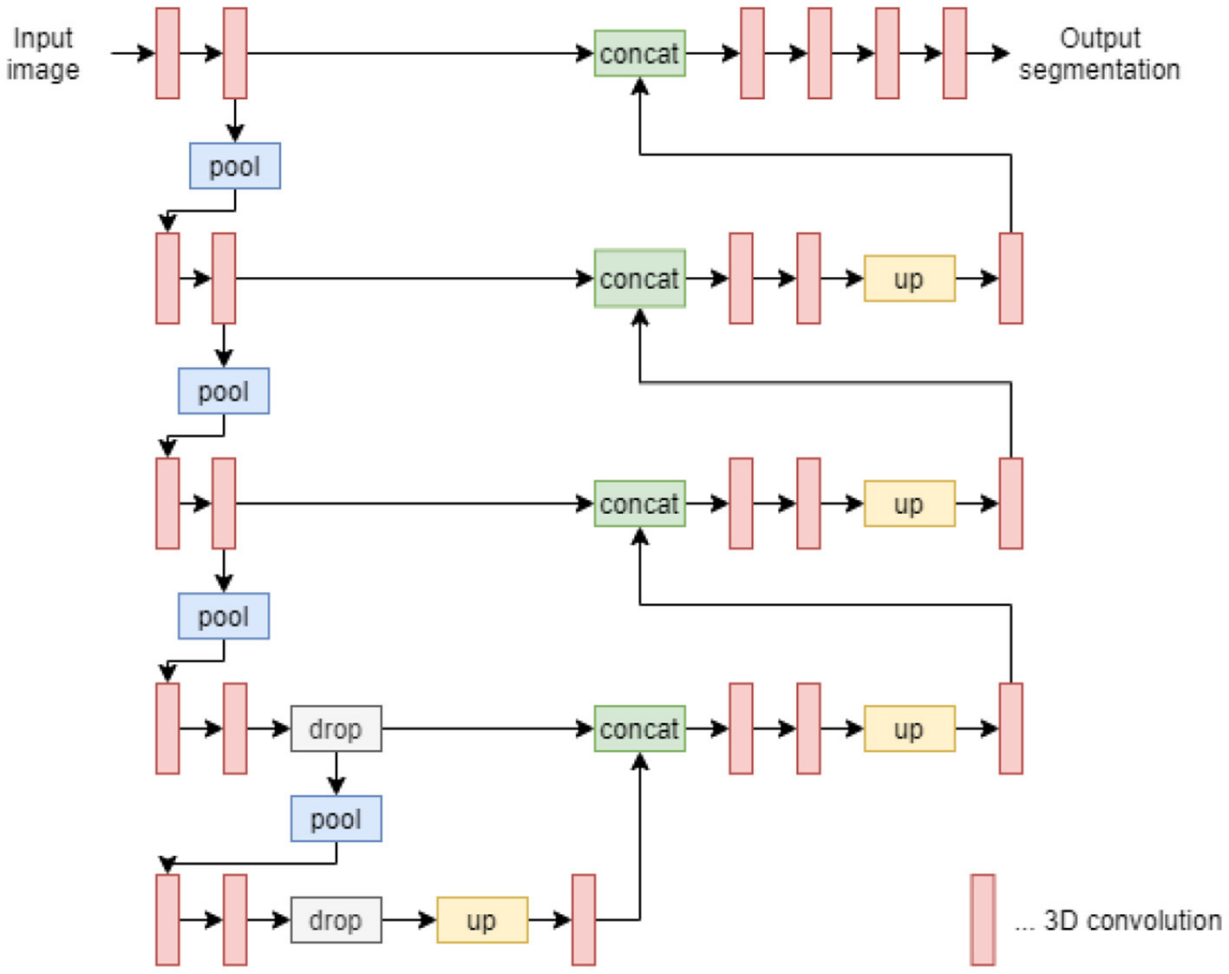
U-Net 3D architecture diagram.

To create a U-Net working with 3D cubes, it is necessary to replace 2D convolution layers with 3D convolution layers, as well as replace 2D max-pooling layers and 2D upsampling layers with their 3D counterparts. In other aspects, the architecture remains the same. A diagram describing the architecture is shown in Figure 2.

### 2.3. Body-Navigation Features

**Figure 3:**
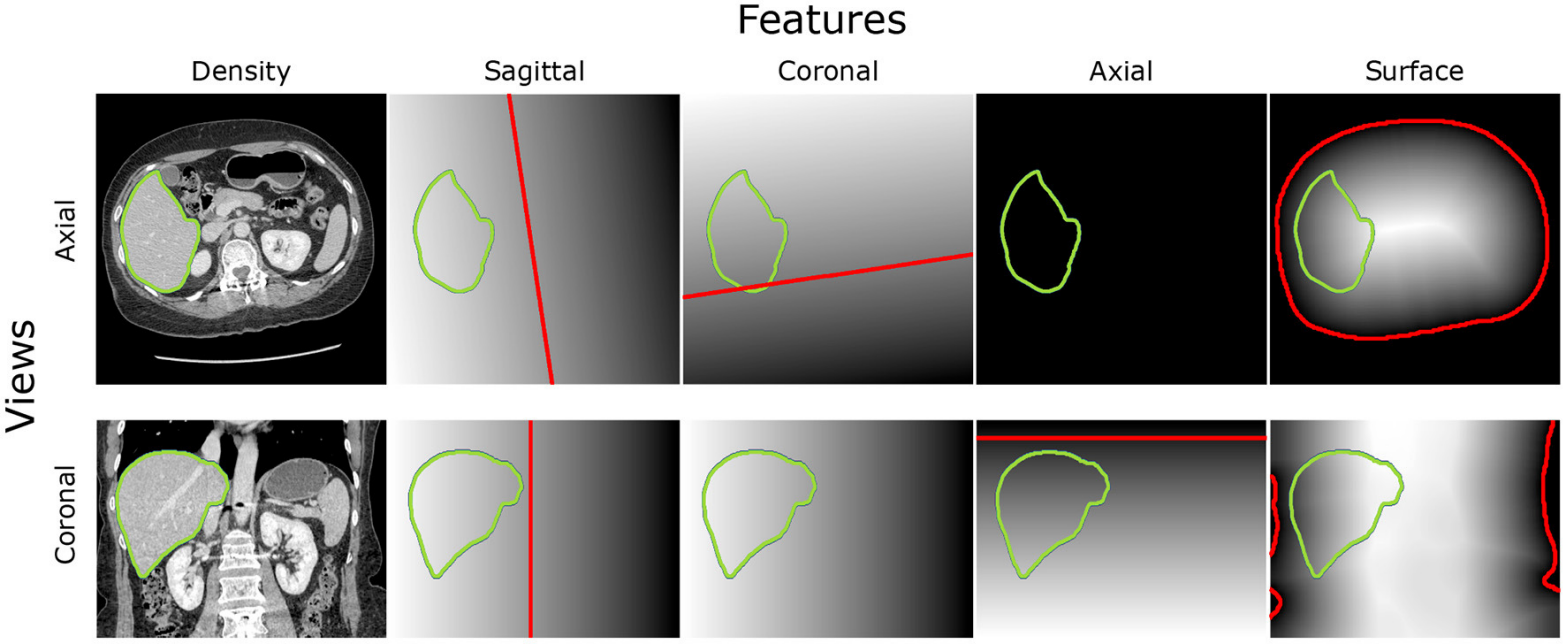
Intensity features and position features based on robustly segmentable structures: The axial view on the middle slice of the CT scan can be found in the top row, while the coronal view is in the second row. The first column is the intensity image. The other columns show the Signed Distance Field (SDF) to the sagittal plane, coronal plane, the axial plane on the bottom level of the heart, and the body surface. The zero level of the SDF is red. The border of the liver is green.

The extraction of the body-navigation features is based on robustly segmentable structures which are accessible to segment, and its position in the body is stable - bones, body surface, and lungs (see Figure 3). We used previously introduced algorithms (Jirik and Liska, 2018) from the bodynavigation package for Python. In addition, we fine-tuned the algorithms for the sake of robustness. The first step is the image resize to a lower resolution for the sake of speed. Generally, for most features, the next step is segmentation, followed by SDF construction.

The *body surface* is extracted by thresholding of filtered image. A used threshold (parameter *TB*) is −300 [HU](Hounsfield Units), and a Gaussian filter does the filtration with a standard deviation (parameter *SB*) of 3 mm.

The detection of the *sagittal plane* starts with the localization of the spine.

Gaussian filter does it with a standard deviation (of 100 mm in the axial (or transversal) plane and 15 mm in the perpendicular directions (parameter *SS*). It is applied on volumetric data thresholded by value 320[HU] (parameter *TS*). Then the center of the body surface and the center of the spine is used to construct a vector that defines the first estimation of the sagittal plane. In the following step, the 2D projection of bones into an axial plane is constructed. Then the mirror image of this projection is prepared. Finally, the symmetry is found by the iterative minimalization of the difference of 2D projection and its rotated and translated mirror image.

The *coronal plane* is the plane perpendicular to the sagittal plane, and it is going through the spine. Thus, the orientation of SDF is given by the position of the center of the body surface.

**Figure 4:**
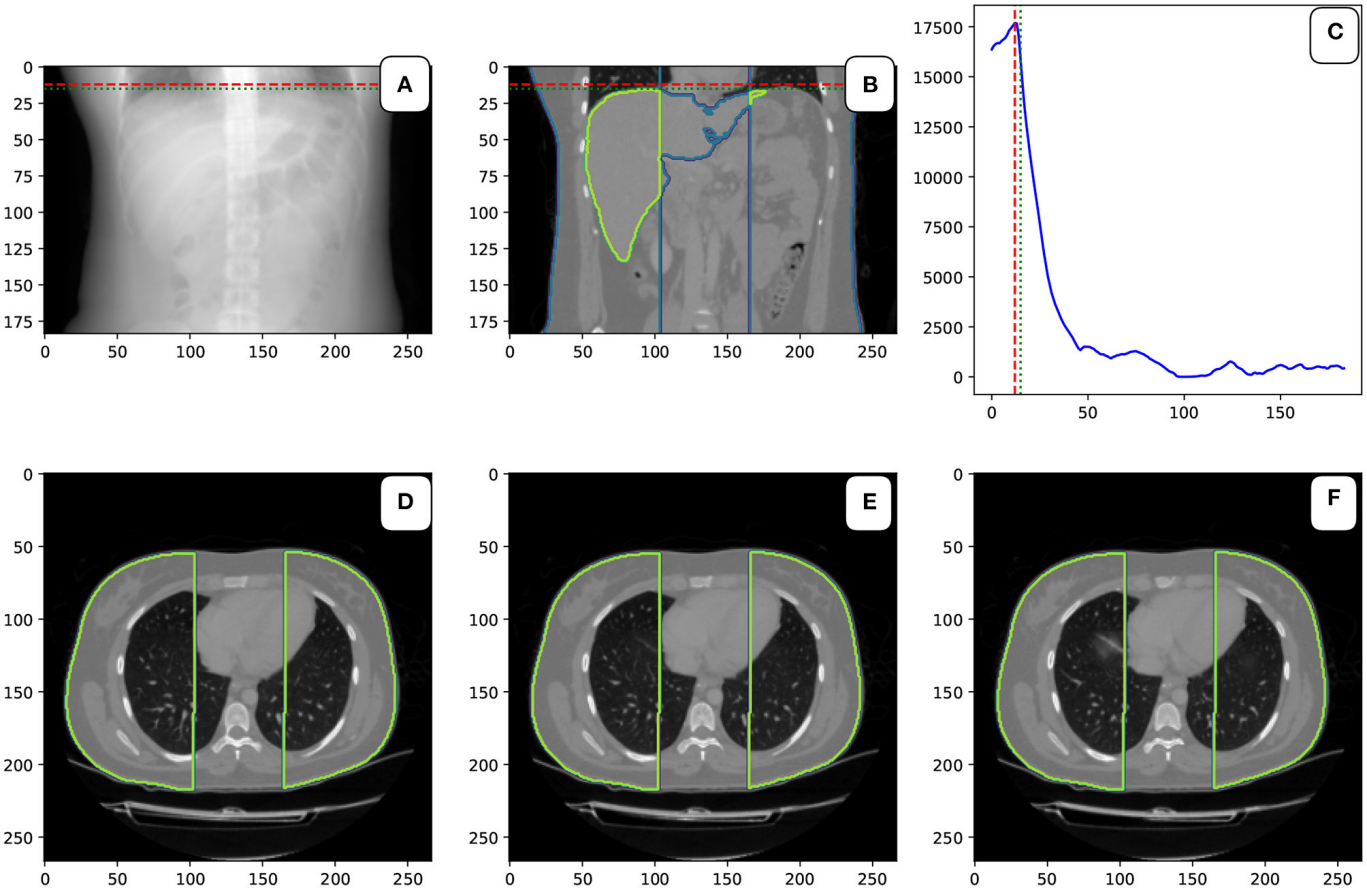
Localization of the top of the diaphragm: Image **(A,B)** in the first row show the CT scan's coronal view. The green and blue delineation show the liver and the mask. The red dashed line is the detected level of the top of the diaphragm. The green dashed line shows the top level of the liver. The image **(C)** in the first row is the plot with the area of the masked cavity in square mm. Images **(D–F)** show three consecutive axial slides of the CT scan. In the middle **(E)** is the slice from the top level of the diaphragm can. On the left **(D)** is the slice above the diaphragm. The right image **(F)** shows the slide under the top level of the diaphragm. A small “shadow” of the diaphragm can be seen in this image.

We introduce the reference point in the craniocaudal axis, located at the top of the diaphragm. The localization of the point is based on the analysis of the series of slides. The internal cavity is defined by the density lower than −200 [HU] (parameter *TLA*). For each slide, the area of the internal cavity was measured. The areas closer than 30 mm to the sagittal plane (parameter *DSA*) and closer than 1 mm to the body surface (parameter *DBA*) are excluded from the calculation. This mask can be seen in the bottom row in Figure 4.

### 2.4. Postprocessing

After performing segmentation using a neural network, we obtain an output image formed by one channel with the same dimension as the input of the neural network. The values of the output image are in the range from 0 to 1. This output is further processed. First, thresholding is performed with a threshold value *Th* = 0.5.

The image is then divided into contiguous areas, and their volume is measured for each of them, given the number of voxels. The area with the largest volume is left in the image, and the others are overwritten into the background.

We use mathematical morphology for further processing of the resulting image. The main reason for including this step was to simplify the boundary of the resulting object and to remove high-frequency noise. In the search for a suitable sequence of morphological operations on the test dataset, the best results were obtained by repeated use of four binary erosions.

## 3. Experiments

To compare the benefits of usage of the body-navigation features in liver segmentation, we designed and implemented two experiments, one using U-Net 2D and one using U-Net 3D. In both experiments, the results of segmentation using the body-navigation features were compared with the results achieved without their use.

Section 3.1 describes the data on which the neural networks were trained and used to validate and test the segmentation model. In section 3.2 we describe metrics used to evaluation of the segmentation results. In section 3.3, we describe the training setup for the experiments, and finally, in section 3.4, we present the results obtained in our experiments using the above approaches. The source code used to perform these experiments is located on the LiverSegmentation repository.

### 3.1. Dataset

For our experiments, the dataset was composed of two public datasets. The *Sliver07* dataset has been created by The Medical Image Computing and Computer-Assisted Intervention Society (MICCAI) for liver segmentation challenge (Heimann et al., 2009). In this dataset, the liver is outlined by a radiological expert in 20 CT scans. The other dataset is *3Dircadb* by Soler (2016), and it was created by Research Institute against Digestive Cancer (IRCAD). It also contains 20 CT scans and liver segmentations made by the radiologist. The number of slices in each series is from 64 to 515. Slice thickness and pixel spacing vary from 0.5 to 5.0 and from 0.54 to 0.87 mm, respectively. By the combination of these datasets, we get 5,777 2D slices and 733,037 3D cubes in total. For our experiments, the dataset was split into three parts—training, validation, and testing. The number of CT slices in training, validation, and testing is 24, 6, and 5, respectively. The entire dataset contains 564,200,960 voxels, while the liver, according to annotations, makes up only 7.72% of images. Segmented classes are therefore significantly unbalanced, so it is appropriate to use a weighted loss function (see section 3.3).

### 3.2. Evaluation Metrics

To compare the results of the individual segmentation methods, we used three commonly used metrics. The first metric is accuracy. This metric expresses the ratio of correctly classified voxels to the total number of voxels in the image. The formula for calculating this metric is shown in the Equation (2), where *TP* is true positives, *TN* is true negatives, *FP* is false positives, and *FN* is false negatives. This metric is often used not only in segmentation but also in classification tasks. However, due to the unbalanced ratio of the number of voxels that contain liver and the total number of voxels, it is not very suitable. To be more specific, the ratio between voxels of the liver and the other voxels is approximately 1:16.816. However, we decided to list accuracy because it is the standard metric.


(2) $$\begin{array}{r} \text{Acc}=\frac{TP+TN}{TP+TN+FP+FN} \end{array}$$

As another metric, we used the Dice similarity coefficient (also known as Intersection over Union), which is more suitable for our segmentation task. It was independently developed by Dice (1945) and (Sørenson, 1948). Dice similarity coefficient measures the similarity between two sets of data *X* and *Y*. In our case, these sets are formed by predicted segmentation and true segmentation. The calculation of this metric is described by the Equation (3).


(3) $$\begin{array}{r} \text{Dice}=2\frac{\left|X\cap Y\right|}{\left|X|+|Y\right|} \end{array}$$

In medical imaging of the parenchymatous organs, the position of the wrongly classified voxel is critical. It is not a big problem if the voxel is adjacent to the segmented organ. However, it is more severe if the voxel far from the liver is classified as a liver. For this reason, we also measured the Hausdorf distance (see Aspert et al., 2002). This metric measures the maximum distance between the surfaces of the actual segmentation and the predicted segmentation. The maximum distance between surfaces $S_{1}$ and $S_{2}$ can be calculated according to the Equation (4) where $d\left(S_{1},S_{2}\right)$ is the distance from surface $S_{1}$ to surface $S_{2}$ and can be calculated with the Equation (5).


(4) $$\begin{array}{r} \text{MaxD}=max\left(d\left(S_{1},S_{2}\right),d\left(S_{2},S_{1}\right)\right) \end{array}$$

(5) $$\begin{array}{r} d\left(S_{1},S_{2}\right)=max_{p\in S_{1}}\left(d\left(p,S_{2}\right)\right) \end{array}$$

### 3.3. Training Setup

For both neural networks (2D and 3D U-Net), the Adam optimizer with a learning rate of 0.01 was used for the training. Weighted binary cross-entropy was used as a loss function, where the weights of the individual classes were adjusted inversely proportional to class frequencies in the input data. In the 2D experiments, the batch size was set to 32 and in the 3D experiments to 4. In all experiments, the networks were trained for five epochs.

### 3.4. Results

**Figure 5:**
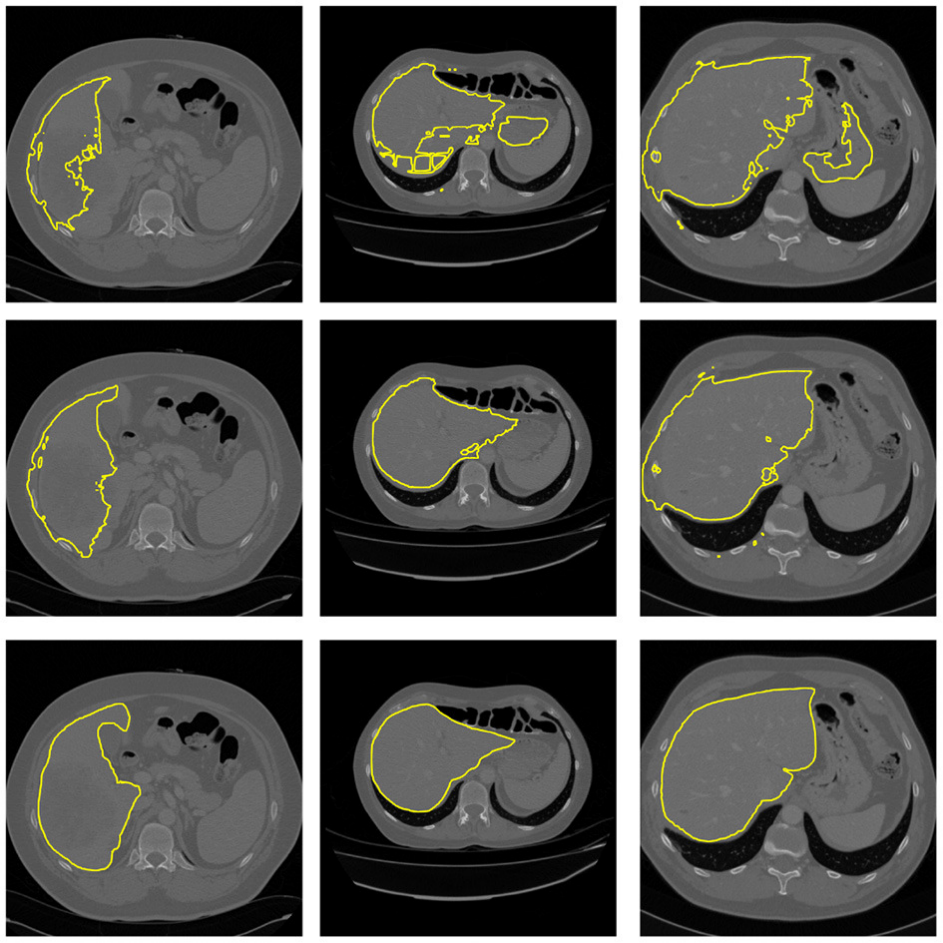
Example of segmentation results from 3D U-Net. **Top**: Segmentation without body-navigation features. **Middle**: Segmentation with body-navigation features. **Bottom**: True mask.

The performed experiments can be divided into two groups—2D and 3D—according to the dimensionality of input data. In both of these groups, we performed the experiment with and without the body-navigation features. The measurements on the validation dataset and test dataset can be found in Table 1. An example of output segmentation is shown in Figure 5.

**Table 1:** Segmentation methods comparison on validation and test dataset.

		**2D model**			**3D model**		
**Image**	**Body-nav**.	**Accuracy**	**MaxD**	**Dice**	**Accuracy**	**MaxD**	**Dice**
3Dircadb-15	No	0.9898	50.8189	0.9094	0.1340	279.6009	0.1025
3Dircadb-16	No	0.9773	52.0738	0.8713	0.1610	305.2199	0.1498
3Dircadb-17	No	0.9773	54.6727	0.8755	0.1610	269.8953	0.1498
Sliver07-16	No	0.9831	37.2269	0.8912	0.1877	316.4408	0.1944
Sliver07-17	No	0.9911	224.5562	0.8361	0.1414	253.8510	0.1158
Sliver07-18	No	0.9849	250.1329	0.7539	0.1359	284.2670	0.1059
3Dircadb-18	No	0.9732	79.9842	0.8892	0.1528	335.1785	0.1356
3Dircadb-19	No	0.9678	71.5489	0.7918	0.1032	406.9116	0.0453
3Dircadb-20	No	0.9851	50.0576	0.8815	0.1032	304.3232	0.0453
Sliver07-19	No	0.9616	118.8727	0.7853	0.1504	274.8742	0.1315
Sliver07-20	No	0.9731	245.2699	0.7165	0.1141	274.7924	0.0660
Avg. value	No	0.9786	131.9546	0.8240	0.1404	300.4868	0.1129
St. dev.	No	0.0092	85.0098	0.0646	0.0250	40.3931	0.0445
3Dircadb-15	Yes	0.9871	34.0230	0.8897	0.9746	100.6067	0.7981
3Dircadb-16	Yes	0.9706	31.3055	0.8118	0.9576	88.0981	0.7201
3Dircadb-17	Yes	0.9780	53.7383	0.8799	0.9675	128.1953	0.8191
Sliver07-16	Yes	0.9818	33.5785	0.8801	0.9545	90.1366	0.7766
Sliver07-17	Yes	0.9931	45.1112	0.8695	0.9633	83.2325	0.7538
Sliver07-18	Yes	0.9928	151.0889	0.8645	0.9565	139.8558	0.6247
3Dircadb-18	Yes	0.9761	41.7075	0.8964	0.9680	93.3783	0.7934
3Dircadb-19	Yes	0.9721	46.9303	0.8146	0.9868	69.3535	0.7698
3Dircadb-20	Yes	0.9817	48.7038	0.8584	0.9872	99.6428	0.7317
Sliver07-19	Yes	0.9693	57.9692	0.8209	0.9624	83.0969	0.7634
Sliver07-20	Yes	0.9829	187.7416	0.7968	0.9860	65.3701	0.8196
Avg. value	Yes	0.9816	75.4617	0.8509	0.9695	94.6333	0.7609
St. dev.	Yes	0.0083	52.1915	0.0353	0.0118	21.4375	0.0528

*All the values are without post-processing*.

Based on measured data, it can be seen that the body-navigation features enhance the quality of segmentation. The effect is stronger in the 3D experiment where the Dice coefficient increased noticeably. In both experimental setups, there has been a significant decrease in the Hausdorff distance. In the 3D setup, the improvement was much more significant once again.

The direct comparison of the 2D and the 3D neural network is inappropriate because, in the 2D setup, the whole slide gives a good idea about the relative position of each pixel in the human body. While using the 3D blocks, there is excellent information about the detail, but the spatial context is lost.

To evaluate the influence of the postprocessing step, the Table 2 contains all the metrics with and without postprocessing. The benefits of postprocessing are most evident in the measurement of the MaxD metric. The cause is the removal of incorrectly segmented small areas that are far from the segmented organ.

**Table 2:** Evaluation of postprocessing in 3D segmentation model.

		**3D model**		
**Postprocessing**	**Body-nav**.	**Accuracy**	**MaxD**	**Dice**
No	No	0.1404	300.4868	0.1129
No	Yes	0.9695	94.6333	0.7609
Yes	No	0.1552	292.2695	0.1237
Yes	Yes	0.9767	50.1791	0.7918

The position features can be generally used not only for liver segmentation. The CT density is not sufficient for the identification of the organ. I.g, the density of the liver is often similar to the density of the heart or spleen. The position of an organ is an important clue in the identification and segmentation process. We believe that there is potential benefit in using our positional feature-based algorithm to segment the other organs and anatomical structures in the abdominal cavity.

In our future work, it would be appropriate to consider the use of a combined segmentation method that would use a 3D convolution network working with local cubes as preprocessing for a 2D convolution network that would process entire 2D slices. Another possible approach would be to extend the input shape of the 3D convolutional network to include several whole 2D slices.

### 3.5. Position-Features Sensitivity

The position features algorithm is based on several parameters. To check the sensitivity to these parameters, we decided to include a test based on the stability of the point in the origin of the 3D space given by the sagittal plane, coronal plane, and the top level of the diaphragm. The default parameter value was multiplied by the parameter value multiplicator *k*. Then the position of the origin point obtained with default parameters is compared to the origin point obtained with the parameter multiplied with *k*. The origin of the coordinance system obtained by *k* = 100% is used as a reference.

**Figure 6:**
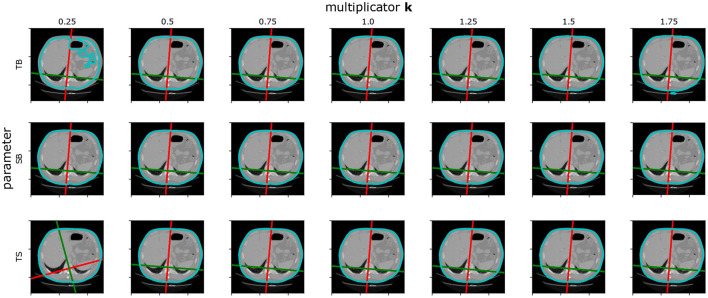
Example of sensitivity of positional features to the algorithm parameters. *k* is the parameter value multiplicator. *SB* and *TB* are parameters sigma and threshold for Body extraction. *TS* is the threshold used in spine extraction. The surface segmentation error can be seen if the parameter *TB* is set to 0.25 of its default value. The sagittal and coronal plane position is lost if the parameter *TS* is set to 0.25 of its default value.

Table 3 shows the mean error for all the investigated parameters over 20 images of the Ircadb1 dataset. It can be seen that the input parameters can be changed in a wide range with no significant impact on the result. The only exception is the threshold used for surface extraction. However, because of the intensity calibration of the CT data and the high contrast between the body and the air, it is easy to set it correctly. The example of the position features extracted with changed parameters can be found in Figure 6.

**Table 3:** Sensitivity of the parameters for the positional features algorithm measured by relative displacement of the origin of the coordinate system: *k* is the default parameter value multiplicator.

	**Error [mm]**						
**k**	**25%**	**50%**	**75%**	**100%**	**125%**	**150%**	**175%**
**Param (default value)**				**Reference**			
SB (3 [mm])	3.3002	2.7002	3.3002	0.0	10.5750	19.7250	24.9750
TB (−300 [HU])	80.2767	39.3823	10.9500	0.0	3.1501	3.9002	3.5253
TS (320 [HU])	77.9674	41.8941	31.0902	0.0	24.7194	42.3552	65.1209
SS (100 × 15 × 15 [mm])	28.8292	22.3974	10.0019	0.0	21.7733	28.1594	31.8916
TLA (−200 [HU])	88.2000	25.4250	0.3750	0.0	0.6750	0.7500	1.2750
DBA (1 [mm])	0.0000	0.0000	0.0000	0.0	0.0000	0.2250	0.2250
DSA (30 [mm])	0.6750	0.6000	0.4500	0.0	2.7000	3.2250	5.1000

*The k = 100% is used as a reference origin point. SB and TB are parameters sigma and threshold for body extraction. TS and SS are parameters for spine extraction. TLA, DBA, and DSA are parameters for axial plane extraction*.

## 4. Conclusion

Our paper presents a method for incorporating position information into automatic liver segmentation performed by a neural network. In addition, we introduced a novel feature for the estimation of the position in the craniocaudal axis. To show the effect of the positional information, we perform experiments utilizing two different data structures. The experiments showed that the position information could significantly enhance the final segmentation quality. Furthermore, the effect is even more substantial if the training data are split into smaller blocks where the spatial context is hidden beyond their borders.

In our future work, we would like to prove the possibilities of using the positional features to segment other organs in the abdominal cavity. Furthermore, we plan to combine both tested approaches and test other possible data processing, especially in the 3D domain.

## Data Availability Statement

Publicly available datasets were analyzed in this study. This data can be found here: https://sliver07.grand-challenge.org/, https://www.ircad.fr/research/3dircadb/.

## Author Contributions

MJ, FH, and VL conceived of the presented idea. MJ modified the positional features from the bodynavigation package, suggested a new diaphragm-based feature, and wrote important parts of the manuscript. FH designed and implemented the 3D processing pipeline and the related experiment, the author of the related manuscript parts, collected the data, performed the annotations, and wrote the section Introduction and data preparation paragraphs. IG provided the consultations on Convolutional Neural Networks, contributed to the sections Introduction and the Conclusion of the manuscript, and made the final proofreading. MZ, VL, RP, and HM provided critical reading and suggested important changes in the manuscript, also provided critical feedback, and helped shape the research, analysis, and manuscript. All authors contributed to the article and approved the submitted version.

## Conflict of Interest

The authors declare that the research was conducted in the absence of any commercial or financial relationships that could be construed as a potential conflict of interest.

## Publisher's Note

All claims expressed in this article are solely those of the authors and do not necessarily represent those of their affiliated organizations, or those of the publisher, the editors and the reviewers. Any product that may be evaluated in this article, or claim that may be made by its manufacturer, is not guaranteed or endorsed by the publisher.
